# Microscopy of the Teeth

**Published:** 1868-09

**Authors:** S. P. Cutler

**Affiliations:** Professor of Chemistry and Microscopy in the Ohio Dental College, Cincinnati. Formerly Professor of Chemistry and Natural Sciences in the Botanic Medical College, Memphis, Tenn.


TO BE CONTINUED.

### MICROSCOPY OF THE TEETH.

BY S. P. CUTLER, M. D., A. E. G., D. D. S.

Professor of Chemistry and Microscopy in the Ohio Dental College, Cincinnati. Formerly Professor of Chemistry and Natural Sciences in the Botanic Medical College, Memphis, Tenn.

*Pus in the pulp cavity of a tooth.* A day or two since a young lady called on me with an aching tooth, the gum much swollen and inflamed about it and several neighboring roots. I extracted one of the adjoining roots, then drilled into the pulp cavity of the decayed and aching tooth. As the drill entered the cavity out came a quantity of pure pus, with considerable force, as though propelled by gas or some other *vis a tergo.*

This soon relieved the sensation of fullness, also the pain. I have often seen similar cases before. I do not mention this circumstance as being anything unusual, as every Dentist has most probably witnessed cases of like character. But here lies, perhaps, the philosophy of a long unsettled or mooted point, but more recently having been regarded as settled, only to be again by this simple circumstance, brought back into the domain of doubt and uncertainty.

In the above case, we find the entire contents of the pulp cavity, the nerve fibres, cellular tissue, fat or cell contents, membranes, blood and vessels all, apparently, converted into genuine pus, unmixed with any impurities, not even the slightest trace of red corpuscles in it. Now, it is generally believed, that pus is a colloid product formed from the blastema or nutrient materials of the fluids of the body. This simple circumstance has opened up new ideas in my mind, whether or not, under all circumstances, pus is a histological element, or something else, or is produced in more ways than one.

In this instance, the entire contents of the pulp cavity were found to be pure, genuine pus. Now, here is a question: were the blood corpuscles contained in this cavity all discolored by oxydation of the hæmatine and chemical action, changing all other contents into pus, or did the red blood in some way make its escape from the cavity through the foramen, or was it, as was once held, discolored blood, that formed part of the pus in this instance, as they cannot be distinguished from pus corpuscles? In this cavity we have a better laboratory than can be found in the system for isolating and circumscribing inflammatory action, resulting in the formation of pus, as here there is but one ingress or egress for fluids, that even may soon be cut off after a certain stage of diseased action.

In the first place, there exists simple stasis of the blood, caused by ennervation, or lessened vitality. This stage is of short duration, as inflammation is sure to follow if not relieved or terminated by dissolution. Soon after congestion follows inflammation and chemical changes begin, and the inorganic soon triumphs over the organic or chemico-vital action, which tendency is downward, pairing off into the binary compounds which exist only as waste material in the organism with few exceptions, as water uncombined, and some minerals as common salt, which, as such, do not form tissue. It would be considered heterodoxical in pathology to say that pus when formed was outside of the organism and beyond its control, further than mere contact, that is it does not make blood or tissue again, and is regarded by the organism as a foreign body or element, and has no essential use only as an adventitious body, which may, in some cases, answer a valuable purpose in the system, by undergoing oxydation when absorbed, thereby sustaining animal heat, or in cases of traumatic injuries serves to shield and protect granulations and inflamed surfaces from whatever cause. In such case it acts as unguents by excluding air, where, resorbed and carried into the circulation in cases of suspended digestion may protect vital fluids and solids by its more ready oxydation.

Again, I maintain that pus is more the result of chemical, than vital action, so called, and is not governed or presided over by the organic or vital forces like other histological elements, and that the true pus corpuscle is dead and not living matter, though when involuted does not act as a poison to the system as putrid matter does, or matter undergoing putrefaction.

That is to say pus is not putrid matter while it remains as such. It is, when exposed to air, broken up much sooner than more vital organizations. Now, whether or not pus is a vital product, or is governed by vital laws while enclosed in positions in the body is, I may say, problematical at least. The fact of its being found in the blood (pyæmia) is an accidental occurrence, and when thrown out from healing wounds serves a valuable purpose for a time, as above stated. Pyohæmia may occur in some cases, but not always; pyohæmia is always a prominent symptom. As I have said, pyogenisis is not fully established yet. Pyorrhagia frequently results in superficial collections. Pus may be coagulated by heat, acids and alcohol. Normal pus consists of two parts pus corpuscles and liquor puris, or watery fluid. There is, however, morbid pus, as pus malignum, ichor sanies, though generally of the same nature. Schneilgue found pus to consist of albumen and water, extractive matter, soda, salts of lime and other salts. We have then plastic material in pus, but it has undergone changes, and is not directed by vital forces--only in part, mainly chemical after the earlier stages, and hence, it cannot be built up into tissue under any circumstances. Vogel maintains that pus may be formed from solid fibrin, after its coagulation. Wedl maintains that it is formed from an exudation by a retrogressive metamorphosis. Others maintain that it is a changed exudation. Mucosine, pyin and casein are maintained by Lehman to be abnormal constituents of pus serum. Many other constituents, as accidental circumstances may be found, such as blood corpuscles, epithelial cells, resinous bile, acids, urea and sugar. The only normal histological element is the cytoid, or pus corpuscles, which measure $\frac{1}{2400}$ of an inch, being a little larger than the corpuscles of blood. They are smaller in abscesses than in wounds, so says Vogel. I might enumerate other characteristics given by authors, but I have given enough for my purpose. The object of this paper is to show that the entire contents of a pulp cavity of a tooth was completely metamorphosed into genuine pus, without any blood colored corpuscles being found in it. In the pulp cavity then we have pus formed of all the elements there is contained without any exudation from any source whatever. Instead we have alone the pulp with its circulation transformed. In such cases, the precise time of the cessation of the circulation cannot be definitely ascertained. At the precise time pus begins to form, after the circulation ceases, is yet unsettled.

I here maintain, that pus is not a vital product, but a post vital result, or a chemical product, and is, consequently, outside of the vital influence, although surrounded by living tisssue, especially in the soft parts. As already stated it may suffer involution, and find its way into the circulation and either be burnt out or excremented.

Does not the above case prove to us that not only organized tissue may be completely metamorphosed into pus, but that red blood corpuscles may also, under favorable circumstances, be decolorized and devitalized into pus corpuscles, and the serum and liquor sanguinis into purin or liquor pyin or pus serum? The reason why blood, under ordinary circumstances, is not converted into pus in the inflamed part is that it is carried out of the part by the blood vessels remaining intact, at least, until their contents are removed. If any of the capillary vessels should suffer transformation, as in the tooth, the blood would have a chance to escape. In the case of the tooth, the blood happens to be arrested in this small prison cell, and all chances of escape are cut off by loss of vitality in the pulp. I deem it a matter of impossibility for all the blood to make its escape fully out of that cavity after once inflamed, even when resulting in death of the pulp, without the formation of pus, which often happens, the foramen being so small that a little swelling is sufficient to mechanically prevent the return of blood in a normal manner from the solid unyielding cavity of a tooth, and so soon as the pressure becomes sufficient to paralyze the pulp then there could be no farther return of the circulation from the pulp cavity. I maintain that the circulation does not depend on any hygrological laws for its normal action, but on vital force residing in the vaso-motors, fibres of the vessels, and polarity of corpuscles all combined.

The fulness felt in the alveolar cavity, in the above case, was no doubt caused by the expansive force of the gases that were being formed in the cavity, after and during the formation of pus, exerting considerable mechanical force through the foramen on to the inflamed and tender tissues of the socket, where it was prevented from making its escape. The discharge of pus gave entire relief, and there is also a reasonable hope of saving the tooth after the subsidence of inflammation.

This same fulness is also felt during the formation of pus in the soft tissues, but in them absorption and condensation, by solution, into the fluids of the adjoining tissues occur, hence relief follows after the pressure is taken off from the sensitive tissues surrounding the abscess. Pus may remain in its seat for sometime without rapid change, though it gradually loses its cytoid character and passes into acid and other formations, resulting in a greater degree of liquefaction, it becomes more fluid and watery, water and gases being the chief products of such decomposition. Such change could not take place freely in the tooth cavity owing to absence of oxygen.

From observations made by the microscope by my friend Lawrence Johnson, on the circulation of smaller animals, he has arrived at the conclusion, that the circulation of the blood depends largely on its own *vis vitæ*, which he attributes to corpuscular polarity, each one acting on those in contact by attraction and repulsion. This idea will readily account for stasis in a tooth after a certain time, as well as in the soft parts. How much in the formation of pus is due to the presence of free oxygen? In the tooth there could be no free oxygen, only what existed in the fluids and tissues of the pulp cavity, as there could be no possible chance for any ingress after inflammation set in, as the gases forming in the cavity, carbonic chiefly, would by their expansive force effectually exclude all ingress, even if the tubules, terminating at the decayed surface were sufficiently open to admit gases or fluids of any kind, as well as the foramen. If any transmission took place at all it must be outwardly, not inwardly, as already stated. I do not fully admit the doctrine of free-cell development, or development without a germ cell in pus, any more than in any other cytoid formation, or any more than I admit the doctrine of *hetero-genesis of the Younger Darwin*, or free development of yeast cells in beer formation. I believe all must have a germ cell to give force and activity by catalysis or some other manner not any better understood. In the tooth cavity spoken of there could be no special germ corpuscles, simply a pathological condition resulting in death of the pulp, and its conversion into pus independent of all surrounding chemical or vital influences, except that of temperature. How long the pus could have remained without further change is undecided.

Is pus a degeneration or regeneration? If formed from blood corpuscles could they ever be revitalized colored discs again? Flesh and vegetable organisms become dissolved in water and afterwards are reconverted into tissue again or differentiated water, forming the chief agent in these metamorphoses. This is disintegration and integration such as take place in the living tissues as waste and repair. The foregoing exegesis may seem somewhat paradoxical to many, though the true solution of the subject is not yet established. One thing is certain, chemical action is the *sine qua non* in pus formation. When a tooth is extracted containing a normal pulp, and exposed to the air, in a day or two the pulp dries up, or evaporates, leaving only the membranes, cells and nerve fibrils, all else escapes through the foramen, but if put into alcohol, the pulp remains full and normal, only whitened. The entire contents are coagulated by alcohol, and will keep a long time, and may be readily sliced up for microscopic objects. When treated with alcohol, and exposed to air it soon darkens, dries up, and cannot again be restored.

Any hydro-carbon as turpentine, balsam and naphthæ will preserve specimens even longer than alcohol, which contains oxygen. Again, pus begins first to form immediately under the seat of decay, where there is exposure of the pulp. We sometimes see the pulp only partially converted into pus.

